# PROMISE: a real-world clinical-genomic database to address knowledge gaps in prostate cancer

**DOI:** 10.1038/s41391-021-00433-1

**Published:** 2021-08-06

**Authors:** Vadim S. Koshkin, Vaibhav G. Patel, Alicia Ali, Mehmet A. Bilen, Deepak Ravindranathan, Joseph J. Park, Olesia Kellezi, Marcin Cieslik, Justin Shaya, Angelo Cabal, Landon Brown, Matthew Labriola, Laura S. Graham, Colin Pritchard, Abhishek Tripathi, Sanober Nusrat, Pedro Barata, Albert Jang, Shuang R. Chen, Rohan Garje, Luna Acharya, Clara Hwang, Amanda Pilling, William Oh, Tomi Jun, Divya Natesan, Chris Nguyen, Deepak Kilari, Michael Pierro, Bicky Thapa, Frank Cackowski, Alleda Mack, Elisabeth Heath, Catherine H. Marshall, Scott T. Tagawa, Susan Halabi, Michael T. Schweizer, Andrew Armstrong, Tanya Dorff, Ajjai Alva, Rana McKay

**Affiliations:** 1 Division of Hematology and Oncology, Department of Medicine, University of California San Francisco, San Francisco, CA USA; 2 Tisch Cancer Institute, Icahn School of Medicine at Mount Sinai, New York, NY USA; 3 Division of Hematology and Oncology, Department of Medicine, University of Michigan, Ann Arbor, MI USA; 4 Winship Cancer Institute of Emory University, Atlanta, GA USA; 5 Moores Cancer Center, University of California San Diego Health, La Jolla, CA USA; 6 Duke Cancer Institute Center for Prostate and Urologic Cancers, Durham, NC USA; 7 Fred Hutchinson Cancer Research Center, Seattle, WA USA; 8 Stephenson Cancer Center, Oklahoma City, OK USA; 9 Tulane Cancer Center, Tulane University, New Orleans, LA USA; 10 Holden Comprehensive Cancer Center, Iowa City, IA USA; 11 Division of Hematology/Oncology, Department of Internal Medicine, Henry Ford Health System, Detroit, MI USA; 12 Department of Medicine, Medical College of Wisconsin Cancer Center, Medical College of Wisconsin, Milwaukee, WI USA; 13 Karmanos Cancer Institute, Wayne State University, Detroit, MI USA; 14 Johns Hopkins Sidney Kimmel Cancer Center, Johns Hopkins University, Baltimore, MD USA; 15 Meyer Cancer Center, Weill Cornell Medicine, New York, NY USA; 16 Department of Medical Oncology & Therapeutics Research, City of Hope Comprehensive Cancer Center, Duarte, CA USA

**Keywords:** Cancer therapy, Cancer genetics

## Abstract

**Purpose:**

Prostate cancer is a heterogeneous disease with variable clinical outcomes. Despite numerous recent approvals of novel therapies, castration-resistant prostate cancer remains lethal. A “real-world” clinical-genomic database is urgently needed to enhance our characterization of advanced prostate cancer and further enable precision oncology.

**Methods:**

The Prostate Cancer Precision Medicine Multi-Institutional Collaborative Effort (PROMISE) is a consortium whose aims are to establish a repository of de-identified clinical and genomic patient data that are linked to patient outcomes. The consortium structure includes a (1) bio-informatics committee to standardize genomic data and provide quality control, (2) biostatistics committee to independently perform statistical analyses, (3) executive committee to review and select proposals of relevant questions for the consortium to address, (4) diversity/inclusion committee to address important clinical questions pertaining to racial disparities, and (5) patient advocacy committee to understand patient perspectives to improve patients’ quality of care.

**Results:**

The PROMISE consortium was formed by 16 academic institutions in early 2020 and a secure RedCap database was created. The first patient record was entered into the database in April 2020 and over 1000 records have been entered as of early 2021. Data entry is proceeding as planned with the goal to have over 2500 patient records by the end of 2021.

**Conclusions:**

The PROMISE consortium provides a powerful clinical-genomic platform to interrogate and address data gaps that have arisen with increased genomic testing in the clinical management of prostate cancer. The dataset incorporates data from patient populations that are often underrepresented in clinical trials, generates new hypotheses to direct further research, and addresses important clinical questions that are otherwise difficult to investigate in prospective studies.

## Introduction

Despite advances in treatment, the median overall survival from onset of metastatic castrate-resistant prostate cancer (mCRPC) remains dismal [1]. In this subset of patients, there is a variable response to currently available therapies, and treatment in these men has not historically been guided by molecular biomarkers. Over the last decade, advancements in genomic sequencing technologies have allowed for a deeper understanding of the molecular complexity of this disease. Many potentially actionable alterations are now identified, fueling biomarker-based clinical trials of novel molecularly targeted agents as well as standard therapies. An important example of genomically tailored therapy in prostate cancer is the utility of Poly-(ADP ribose) polymerase (PARP) inhibitors in patients whose tumors harbor Homologous Recombination Repair (HRR) defects. The demonstrated clinical efficacy of olaparib [2] and rucaparib [3] in metastatic CRPC with HRR defects and their subsequent approval by the US Food and Drug Administration (FDA) marked an important milestone in the implementation of precision medicine for this disease.

The approvals of PARP inhibitors make it imperative to obtain somatic sequencing for all men with advanced prostate cancer. In addition, germline testing is also recommended for all men with metastatic prostate cancer per National Comprehensive Cancer Network guidelines, and in selected men with localized disease based on clinical risk and histologic subtypes as well as family history [4]. In fact, germline mutations in HRR genes exists in ~5% of localized prostate cancer [5]. In addition, multiple guideline panels recommend genomic sequencing for all patients with advanced prostate cancer. With this shifting paradigm and advancements in technology, there will be a rise in availability, breadth, and scope of genomic data derived from CLIA-based testing platforms. Linkage of such data with granular patient outcomes can be leveraged to improve our understanding of the molecular mechanisms that lead to variable clinical outcomes across prostate cancer disease states over time, identify additional subgroups of patients whose tumors are more vulnerable to specific therapies, and help guide decisions of optimal sequencing of therapies in prostate cancer.

The Prostate Cancer Precision Medicine Multi-Institutional Collaborative Effort (PROMISE) is a consortium of academic cancer centers with the goal of better defining the clinical-genomic features across the entire prostate cancer disease spectrum. Herein, we outline the rationale, design, and objectives of our multi-institutional, retrospective clinical-genomic database of advanced prostate cancer patients that addresses this important need.

## Molecular landscape of advanced prostate cancer

**Fig. 1:**
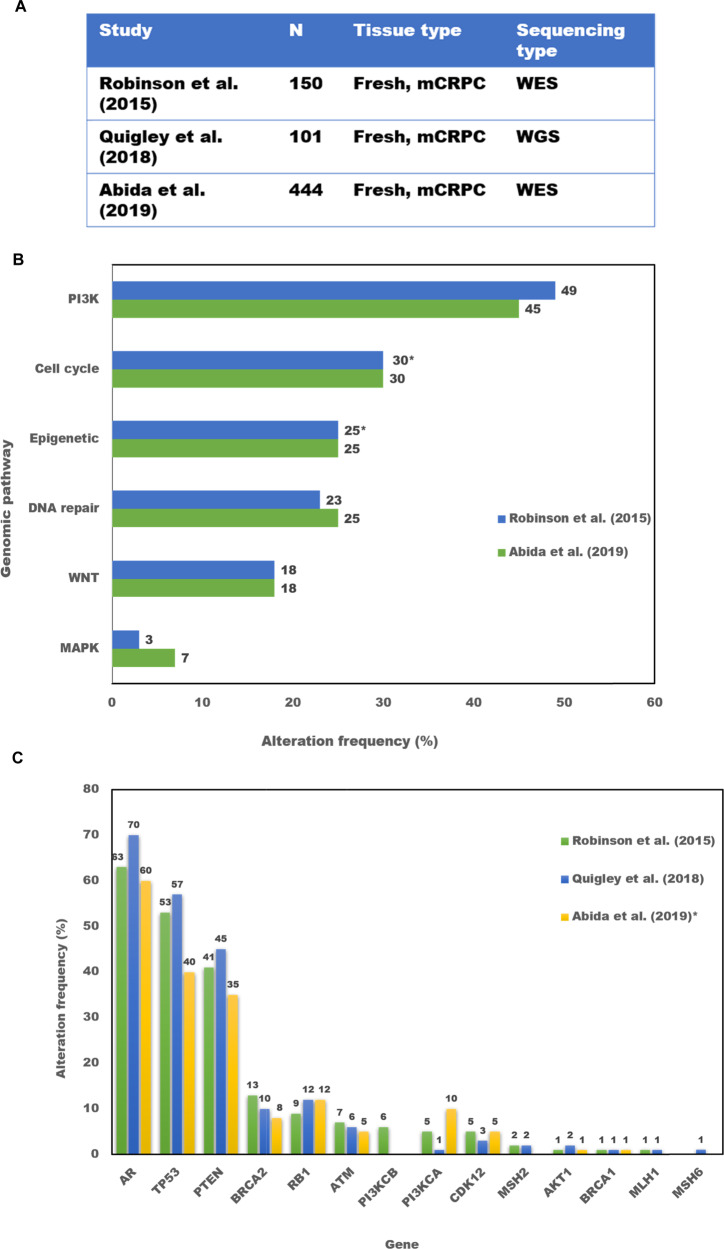
Large-scale genomic sequencing analyses of mCRPC. **A** List of three prospective genomic analyses of mCRPC samples. **B** Distribution of non-androgen-receptor, actionable genomic pathway alterations. **C** Distribution of commonly expressed and actionable genomic alterations. *Represent estimates based original article.

Large-scale genomic analyses of metastatic prostate tumors have demonstrated a high frequency of germline and somatic alterations in several cancer-specific genes that are actionable or currently being investigated as candidate predictive biomarkers (Fig. 1) [6–9]. Some are more commonly seen in localized disease such as *SPOP* mutations and ETS family gene fusions [10]. The most common gene alterations enriched in CRPC, compared to earlier disease states, include *AR* and *TP53*, which are present in >50% of cases [6, 9]. Other commonly affected pathways with important clinical and therapeutic implications include the PI3K pathway genes such as *PTEN-AKT*-*mTOR*, and HRR genes such as *BRCA1/2*, *ATM* which are altered in ~45%, ~25%, ~7-10% of CRPC cases, respectively [6, 8]. Additional altered genes in CRPC include those involved in the cell cycle (~30%) such as *RB* loss, *CDKN1B*, and *CCND1*; epigenetic regulator genes (~25%) such as *KMT2C*, *KMT2D*, and *CHD1*; WNT pathway genes (~15%) such as *APC* and *CTNNB1*, *CDK12* (~7%); and MAP kinase pathway genes (~5%) [6, 8, 9]. These alterations in the context of drug development and clinical decision-making are discussed in further detail in the next section. Many of these alterations occur concurrently, and based on prior reports, about 65–85% of analyzed CRPC tumors harbor potentially actionable alterations beyond AR, defined as the ability to predict response to an available drug based on existing preclinical data [6]. Structural variants in the non-protein-coding regions also have a potential impact on the activity of important regulators of cancer progression [9]. A recent whole-genome sequencing analysis, the first of its kind in CRPC, identified that 81% of cases harbored amplification of a putative enhancer of *AR*, which may drive androgen resistance [9, 11]. Structural variants were also demonstrated near the *MYC, TP53, CDK12, FOXA1*, and *BRCA2* genes with potential biological and clinical implications.

In metastatic hormone-sensitive prostate cancer (mHSPC), treatment options have rapidly expanded, yet decision-making is based mainly on clinical factors without integration of genomic biomarkers. A recent study describes potential biomarkers of disease aggressiveness [7]. Specifically, alterations involving AR, TP53, cell cycle, and MYC pathways predict for a shorter time to castration resistance, while alterations in *SPOP* gene and WNT pathway are associated with lower rate and longer time to castration resistance [7]. Furthermore, in tumors with high-volume (≥ 4 bone metastases and/or visceral metastasis) mHSPC, higher fraction of alterations indicative of genomic instability, and alterations involving cell cycle, epigenetic regulation, and NOTCH pathways are seen. Thus, the development of a clinical-genomic model for both mHSPC as well as CRPC represents a major unmet medical need, for which a large number of patients are needed with careful clinical and genomic annotation.

## Implementation of precision medicine in prostate cancer

Our understanding of the molecular landscape of advanced prostate cancer provides a platform for the development of targeted therapies, and for optimizing therapeutic combination strategies and sequencing. Excitingly, intense investigation is already taking place to leverage this knowledge and expand therapeutic options for men with advanced prostate cancer. Increasingly, genomic sequencing is also becoming a pre-requisite for clinical trials, thus availability of routine genetic testing becomes imperative to ensure patients have access to biomarker-driven therapies and clinical trials.

Approximately 20–25% of men with mCRPC have germline or somatic alterations involving HRR genes, such as *BRCA1/2* and *ATM*, which may predict for sensitivity to PARP inhibitors and platinum agents [12]. Large clinical trials such as PROfound and TRITON2 have led to the FDA approval of two PARP inhibitors, olaparib and rucaparib, respectively, in men with mCRPC that harbor deleterious somatic or germline mutations in HRR genes [2, 3]. In May 2020, olaparib was approved for men with HRR gene-altered CRPC while the rucaparib approval was restricted to patients with tumors harboring alterations in *BRCA1/2*. Despite these approvals, many questions remain about the relevance of PARP inhibitors in patients harboring non-*BRCA* HRR genes. Many of these questions can be answered with a real-world database such as PROMISE. In addition to PARP inhibitors, exceptional responses to platinum-based chemotherapy have also been observed in *BRCA2*-mutant CRPC cases [13, 14]. However, not much is known in this population about the utility of platinum agents and PARP inhibitors in combination or as sequential therapy. Other HRR gene targets under clinical development include ATR inhibitors and ATM inhibitors. Of specific interest within the HRR pathway is *CDK12* loss, which has shown to correspond with high focal tandem duplications and high neoantigen burden, potentially sensitizing *CDK12*-altered tumors to immune checkpoint blockade [15–17]. Thus, knowledge from real-world datasets on the appropriate sequencing and outcomes of taxane chemotherapy with PARP inhibitors in less common subgroups of men with mCRPC such as *ATM* mutations or less common HRR gene alterations is needed.

About 45% men with mCRPC harbor pathogenic genomic alterations within the PI3K pathway. The majority of alterations involving this pathway occur in the *PTEN* gene (~40%), which has previously proven to be a difficult target with single agents and is associated with poor prognosis [18]. Preclinical data suggest that PTEN/PI3K and AR pathways have reciprocal crosstalk, thus targeting both pathways in combination may enhance therapeutic efficacy [19]. A phase II study of abiraterone acetate and AKT inhibitor, ipatasertib, showed a clinical benefit particularly in *PTEN-*deficient mCRPC [20]. In the primary analysis of the phase III study of this combination (IPATential150, NCT03072238), combined AR and AKT blockade provided an improved progression-free survival in patients with mCRPC with *PTEN* loss, compared to AR blockade alone [21]. Another target within this pathway is *AKT1* gene, which occurs in about 1% of mCRPC patients. AKT inhibitors exhibit clinical activity in AKT-mutated breast cancer and other solid tumors [22] and are currently in development for mCRPC (NCT04087174). Other alterations of interest in this pathway involve *PIK3CA*, *PIK3C2B*, *BRAF*, *MAP2K1*, and *KRAS* genes. Recently the first PI3KCA inhibitor, alpelisib, was approved for PIK3CA-mutated breast cancer [23], a development that can inform future clinical trial designs and treatment options for advanced prostate cancer as well.

About 3–5% of patients with CRPC also have evidence of DNA mismatch repair deficiency (dMMR) and/or microsatellite instability (MSH)-high that are found through immunohistochemistry to evaluate for loss of MMR protein and/or DNA sequencing [24]. These patients tend to have tumors with a higher tumor mutational burden (TMB), predicting response to immune checkpoint blockade [25–28]. In 2017, based on data from five multi-cohort, single-arm studies of MSH-H or dMMR advanced solid tumors [29, 30], pembrolizumab received a tumor-agnostic approval for patients who progressed on prior treatment and did not have satisfactory alternative treatment options. Although this study marked the first time in oncology drug development that a therapy was approved based on a specific biomarker irrespective of tumor histology, very few patients with prostate cancer were enrolled. Furthermore, it is not clear whether routine testing of dMMR and/or MSI is being performed in most clinical practices to identify this subset of prostate cancer patients. More recently, tumor-agnostic pembrolizumab approval was expanded to include tumors with high TMB [31].

In addition to potential actionable alterations discussed, multi-institution clinical trials such as Targeted Agent and Profiling Utilization Registry (TAPUR, NCT02693535) by American Society of Clinical Oncology highlight the aims of understanding the safety and efficacy of novel targeted agents in advanced prostate cancer and other malignancies. In addition, large registries of real-world data are being built to identify novel targets. The American Association of Cancer Research (AACR) has implemented Project GENIE (Genomics Evidence Neoplasia Information Exchange), which consist of real-world data among 19 leading cancer centers in the world. Despite the robustness of these pan-cancer platforms, the nuances of different clinical states of prostate cancer will not be captured. To complement these efforts, our consortium was developed to leverage the wealth of existing clinical-genomic data to expand our understanding of prostate cancer biology and to improve current therapeutic approaches. In the remaining sections, we highlight some key clinical questions that are present in routine practice to provide context into the design, structure, and objectives of PROMISE.

## Current challenges in prostate cancer and potential solutions

The last two decades have seen significant new advancements in the treatment of metastatic prostate cancer. Much wider availability of next-generation sequencing (NGS) panels has made it possible to individualize treatment options for a subset of prostate cancer patients with specific tumor markers or alterations. These developments have created a plethora of new questions, many of which may be answered though accumulation of clinical and genomic data in a “real-world” dataset. The following examples highlight current challenges in the treatment of advanced prostate cancer as well as potential solutions and strategies to overcome barriers to the realization of the true promise of precision medicine in prostate cancer.

### Sequencing of therapies in prostate cancer

With the evolution of multiple novel treatment options in mCRPC, including novel hormonal agents and PARP inhibitors, as well as the emergence of novel therapeutic classes such as PSMA-targeted radioligand therapies, there is increasingly a wealth of options to offer patients in a disease space where only a couple of decades ago there were relatively few [32, 33]. In this setting, the questions about appropriate sequencing of therapies become especially relevant, especially as most patients will not be able to receive all available treatment options and decisions of which therapies to prioritize earlier as opposed to later in the disease course must be made by the treating physician. For instance, in molecularly selected patients able to receive PARP inhibitors, what is the most optimal way to sequence these agents with taxane chemotherapy and should the sequencing of therapies be impacted by mutation status? The PROMISE clinical-genomic platform can help to generate initial data on potential positive and negative predictive biomarkers to guide these types of therapeutic questions.

### Novel treatment paradigms in mHSPC and impact on subsequent treatment options

As novel treatment options are also introduced earlier in the disease course of prostate cancer, such as novel androgen receptor signaling inhibitors (ARSIs) [34–37], the natural history of many patients with mCRPC progressing on these therapies earlier in the disease course may also be altered. Do patients who develop castrate-resistant disease while being treated with ARSIs in the hormone-sensitive space subsequently have more aggressive and rapidly progressing CRPC? Do these patients subsequently respond to the standard of care treatment options available for mCRPC similarly to prior populations of patients not treated with these agents in the hormone-sensitive setting? Are these patients more likely to develop treatment-emergent neuroendocrine or small cell prostate cancer [38]? It is incumbent on the prostate cancer research community to better understand this natural history in light of new treatment paradigms. In this dynamic and rapidly changing treatment space much of the available clinical trial data used to guide treatment decisions may reflect a very different patient population than the patients being treated now. Serial cell-free DNA (cfDNA) is a cutting-edge solution to temporally understand therapeutic resistance mechanisms and is expected to be widely adopted in the future. Therefore, the vast genomic data gathered for each patient will be invaluable in our efforts to answer questions related to treatment exposure and acquisition of resistance.

### Natural history and treatment options for novel molecular subtypes in prostate cancer

As treatment for prostate cancer is increasingly individualized and new biological subsets are identified through increased use of NGS, it is also important to better define the natural history of these patient populations. The molecular characterization of exceptional responders and non-responders to standard of care therapies will help better define molecular predictive biomarkers. In addition, as of 2020 there are now two agents, olaparib and rucaparib, specifically approved for the treatment of mCRPC with HRR alterations [2, 3] as well as multiple clinical trial options for patients with advanced prostate cancer and various genomic alterations. This is particularly important since response to standard of care treatments will serve as a benchmark for the efficacy of novel therapies that specifically target these genomic alterations. These questions need to be answered in order for precision medicine to truly fulfill its promise. More individualized treatment, implying the division of the broad diagnosis of prostate cancer into ever smaller molecularly defined subsets, will increasingly help guide the rational design of clinical trials and therapy selection. One early example of this approach is the IND.234 trial (NCT03385655) from the Canadian Clinical Trials group that uses a cfDNA platform to select therapy in prostate cancer. The improved understanding of these molecularly defined subsets through hypothesis-generating retrospective studies can help improve the design of biomarker-driven clinical trials of prostate cancer.

### Impact of race/ethnicity or socioeconomic status on prostate cancer treatments and outcomes

Many studies focusing on characterizing the molecular alterations in prostate cancer have included a very limited number of ethnically diverse subsets of patients. Understanding how race/ethnicity impacts genomic profile and response to therapy is critical to bridging the disparities gap in men with prostate cancer. Real-world data from this platform can fill this gap. Black men remain underrepresented in phase 3 trials in prostate cancer despite being disproportionately impacted by lethal prostate cancer [39] and despite evidence of perhaps superior outcomes when treated with sipuleucel-T or docetaxel [40, 41]. Do these factors impact the diagnostic decisions made, such as the choice of imaging in advanced prostate cancer [42]? Are certain treatment options chosen preferentially over others and does this create healthcare inequities? Among the treatment options chosen, do certain options work better for certain subsets of patients? Such questions are difficult to answer with clinical trials which historically underrepresent many of the populations in question [43]. However, it is absolutely vital to answer these questions in order to help us improve the care of all patients with advanced prostate cancer. The collection of germline data for the patients in a real-world dataset will also help to both understand the landscape of somatic and germline mutations in underrepresented patients and the general availability of germline testing in this patient population [44, 45].

## Utility of real-world data and the unmet need for the PROMISE consortium

All of the above clinical questions and scenarios in advanced prostate cancer illustrate the importance and potential utility of a large and well-maintained real-world database that serves as a repository for clinical and genomic data of prostate cancer patients. There are several pertinent reasons necessitating such a database as an important clinical research tool in advanced prostate cancer.

Prospective randomized clinical trials remain the gold standard in clinical cancer research and shape the current standard of care. While prospective clinical trials capture data on efficacy, observational studies can help to better understand effectiveness of the treatments in the real-world setting. Importantly, most prostate cancer patients do not get their cancer treatment as part of a clinical trial. A recent study estimated that only about 8% of oncology patients enroll in a clinical trial [46]. Consequently, the experiences and treatment outcomes of most prostate cancer patients are not systematically recorded or analyzed. In addition, most clinical trial datasets do not capture the entirety of patient experience from diagnosis until final treatments, focusing rather on one specific treatment outcome, and thus provide only a limited window into the outcomes of patients in the real-world setting. Retrospective studies of real-world patient datasets that include well-annotated clinical and genomic data can shed light on important questions that may be difficult to address in prospective clinical trials.

The inclusion of underrepresented minorities in this dataset is one of the important missions of this consortium. Data from retrospective series can more extensively capture clinical outcomes and other information of more diverse populations, including minority populations underrepresented in clinical trials. The low participation rates of patients who identify as underrepresented minorities in clinical trials of prostate cancer have been well documented and remain a significant challenge in clinical research and the conduct of trials [43]. The reasons for this are complex and multifactorial but it remains a significant challenge that is yet to be addressed. The selection for this consortium of diverse clinical sites representing different geographic regions of the United States that serve unique underrepresented patient populations is certainly an important step toward bridging the cancer genomics disparities gap in prostate cancer. Many of the initially proposed projects focus on questions related to racial disparities.

It is also important to keep in mind that although randomized clinical trials remain the gold standard for generating clinical data, many relevant clinical questions are initially asked in hypothesis-generating retrospective studies. Important descriptive data of the natural history of disease in these specific subgroups can be obtained through retrospective chart review. Many important prognostic and predictive markers and biomarkers of interest can initially be identified through analyses of real-world data and are subsequently validated prospectively. Furthermore, in contrast to prospective clinical trials where treatment options are prespecified, retrospective observational studies can also attempt to capture clinical decision-making, such as decisions made based on the results of genomic testing. In databases with large numbers of patients and well-annotated clinical and genomic data, important insights can be gleaned from this approach.

As treatments are increasingly individualized for molecularly selected subsets of patients with mCRPC, the careful collection of genomic data in large real-world cohorts can also help shed light on tumor biology and help advance questions that produce important validation studies. The availability of multiple tissue or blood samples from the same patient can also be particularly instructive. This information can help better define the evolution of molecular alterations or other biomarkers in the tumor throughout the course of disease progression. The understanding of such changes obtained from serial sampling of either repeat solid biopsies or of repeated cfDNA sampling can also help define the changes in the tumor that happen during progression on specific treatments. Further important comparisons can be drawn in the molecular alterations and other biomarkers detected in solid biopsies and cfDNA in the same patient.

Overall, our long-term goal is to develop a large real-world dataset collected from institutions including both academic and large community-based hospital systems, which by definition will be both heterogeneous and also more representative of real-world trends. Clinical and genomic information from many patients who are unable or unwilling to be treated on a clinical trial due to clinical, logistical or other reasons will also be included. This will make potential findings more generalizable relative to the data obtained from carefully selected clinical trial populations. The patient population included in a real-world dataset will also reflect the diversity of treatment options and strategies employed, diagnostic and genomic testing used, and the diverse patient populations across the different geographic regions.

## Development of PROMISE, its aims, and the inclusion of a dedicated bio-informatics committee

**Fig. 2:**
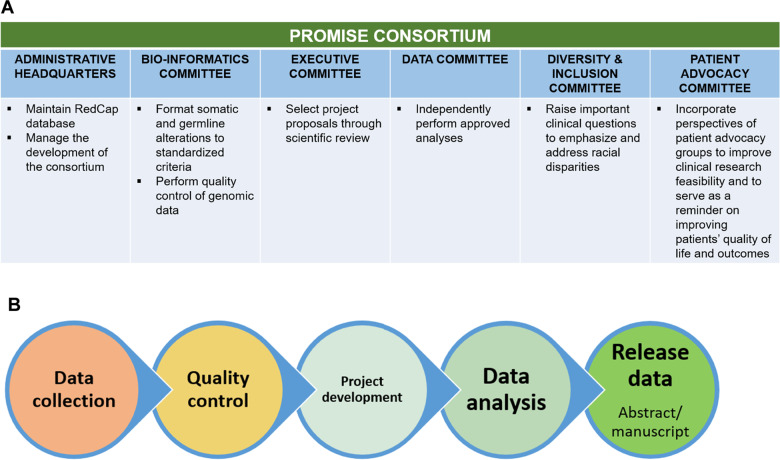
PROMISE consortium structure and workflow. **A** Structure consisting of administrative headquarters, bio-informatics committee, executive committee, data committee, diversity & inclusion committee, and patient advocacy committee. Responsibilities of each committed listed. **B** Project workflow from data collection to release of data in abstract/manuscript form.

The PROMISE consortium was formed with the recognition of the significant clinical and research needs of linking clinical and genomic data to outcomes, in order to better inform treatment decisions and outcomes for patients with advanced prostate cancer. Data collected in this consortium will address significant knowledge gaps that come about as the underlying biology of prostate cancer becomes better understood and multiple novel agents and classes of drugs emerge as treatment options. The structure of the PROMISE consortium including all of the committees and the flow chart of data analysis are shown in Fig. 2.

PROMISE is currently a consortium of 16 academic institutions with the common goal of understanding the real-world data in patients with advanced prostate cancer. The goal is to expand the reach of this database to include academic institutions and community practices. The consortium has two main aims:

(1) To establish a large, diverse, inclusive, and well-annotated repository of completely de-identified clinical and genomic patient data that is linked to clinical characteristics and disease-related outcomes.

(2) To investigate prognostic and potentially predictive biomarkers in patients with advanced prostate cancer treated with approved therapies including genomic, transcriptomic, and proteomic biomarkers.

Inclusion criteria for patients in this dataset consist of having advanced prostate cancer with either mHSPC or castrate-resistant prostate cancer (CRPC). In addition, patients should have undergone prior germline and/or somatic tumor molecular profiling studies including, but not limited to, one or more of the following blood or tissue-based assays: tumor DNA sequencing (e.g., cell-free circulating tumor DNA or tumor tissue sequencing), germline DNA sequencing and transcript profiling studies (e.g., RNA-seq, qRT-PCR). In total, consecutive patients with available clinical and genomic data at each site will be included to limit selection bias. Clinical and treatment data is collected from the time of initial prostate cancer diagnosis (Table 1). All data are maintained in a secure RedCap database and is completely deidentified without any protected health information. Genomic information entered into the database from both solid tissue and liquid biopsy testing and inclusive of both somatic and germline alterations are formatted to standardized criteria and reviewed by the bio-informatics committee. We will only include the entries that have all available data related to clinical outcomes and genomic sequencing.

**Table 1:** Examples of clinical and genomic data collected in the PROMISE consortium.

Clinical data elements				Genomic data elements		
Demographics	Disease characteristics	Lab values	Treatment characteristics	Pathology status	Gene information	Class of alteration
Age	Prior prostatectomy	PSA	ECOG PS	Specimen source	Type of sequencing test	Copy number variation
Race	Histology	Hemoglobin	Systemic therapies	Specimen date	Gene name	Structural variant (SV)
Veteran status	Gleason score	Platelets	Duration of Treatment	MSI	Somatic vs germline	Single nucleotide variant (SNV)
Marital status	Stage at diagnosis	ANC/ALC	Imaging obtained	MMR	Type of alteration	Insertion/Deletion
Smoking status	De novo metastasis	Alkaline phosphatase	Time to progression or death from diagnosis, metastatic disease, and CRPC	PD-L1	Zygosity	Allele frequency (for cfDNA)

## PROMISE consortium institutions, data acquisition plan, and mission statement

**Fig. 3:**
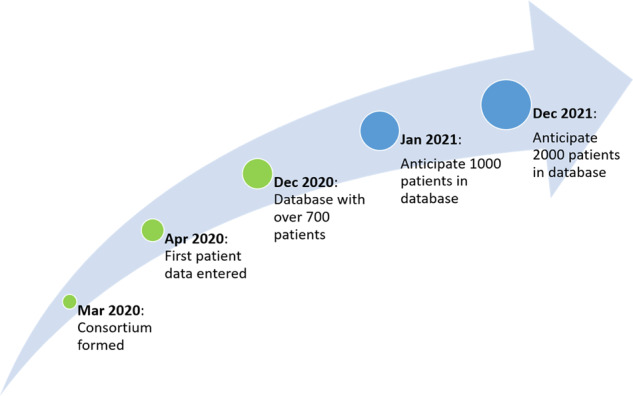
Project milestones and future timeline. The PROMISE consortium was formed in March 2020. At the end of 2020, we collected clinical data of over 700 patients. We anticipate clinical data collection of 2000 patients by the end of 2021.

The PROMISE consortium participating institutions are listed in Table 2. The consortium was formed in the first half of 2020 and data entry to the consortium has since risen steadily as sites have come onboard and over 1000 patient records are anticipated by early 2021 (Fig. 3). Genomic data are being audited for accuracy at each institution.

**Table 2:** Current Institutions Participating in the PROMISE Consortium.

Institution name	Location
City of Hope	Duarte, CA
Duke Cancer Institute	Durham, NC
Emory University	Atlanta, GA
Henry Ford Health System	Detroit, MI
Icahn School of Medicine at Mount Sinai	New York, NY
Johns Hopkins University School of Medicine	Baltimore, MD
Karmanos Cancer Center	Detroit, MI
Medical College of Wisconsin	Milwaukee, WI
Tulane University	New Orleans, LA
University of California San Diego	San Diego, CA
University of California San Francisco	San Francisco, CA
University of Iowa	Iowa City, IA
University of Michigan	Ann Arbor, MI
University of Oklahoma	Oklahoma City, OK
University of Washington	Seattle, WA
Weil Cornell Medical Center	New York, NY

The mission of the PROMISE consortium is to investigate the outcomes of patients with advanced prostate cancer who have genomic and molecular profiling information and therefore to bridge the knowledge gap between real-world genomic data and clinical outcomes in patients with advanced prostate cancer. Consortium structure will also provide significant opportunities for mentorship of junior investigators and trainees. The consortium will plan to pursue multiple projects addressing specific questions which are proposed by the participating site investigators and selected through scientific review by the executive committee with priority given to projects proposed by junior investigators and trainees. Given the important potential contribution of evaluating outcomes in a more diverse population than that represented in clinical trials, a subcommittee for diversity and inclusion has been created with a plan to implement strategies to increase diversity if the database fails to maintain a balanced population.

## Future directions

In a short period of time, we have gathered vast amounts of clinico-genomic information to answer important clinical questions. We are looking forward to expand our database to include many more academic institutions and community practices to make our patient population more representative of the real-world setting. In addition, once the database becomes robust for large-scale analyses, these data may be made publicly available for future research, following efforts such as cBioportal. Lastly, there is a long-term plan to develop infrastructure for blood- and tissue biorepository to perform molecular analyses.

## Conclusions

Precision medicine holds significant promise for helping to individualize treatment approaches for patients with advanced prostate cancer and to help understand the underlying mechanisms of this heterogeneous disease. The PROMISE consortium is an important new collaboration among leading academic centers in the United States that aims to bridge the gap between clinical and genomic information for patients with metastatic prostate cancer and available genomic data from commercial and institutional assays. The well-annotated, de-identified patient data collected for this consortium will be leveraged to help answer specific questions and guide projects proposed by the consortium members. Genomic data entered into the database will be vetted by a bioinformatician and individual projects approved by the executive committee as part of the consortium structure. This organized approach will help to address important questions at the intersection of clinical and genomic data that are most optimally addressed using a real-world dataset. It is our hope that this approach will eventually help to improve outcomes for patients with advanced prostate cancer.
